# A Case of Bilateral Carotid Body Tumors Requiring Surgical Resection: The Importance of Physical Exams

**DOI:** 10.7759/cureus.71082

**Published:** 2024-10-08

**Authors:** Pranav Balakrishnan, Taylor Adkins, Matthew Krantz, James C Kitchen

**Affiliations:** 1 General Surgery, Marshall University Joan C. Edwards School of Medicine, Huntington, USA; 2 Vascular Surgery, Marshall University Joan C. Edwards School of Medicine, Huntington, USA

**Keywords:** carotid artery surgery, carotid body tumor, carotid surgery, comprehensive physical exam, paraganglioma

## Abstract

We present the case of a 61-year-old man who presented to his primary care provider for a routine visit and was found to have a pulsatile mass in his right neck. This case report shows the importance of a thorough physical exam during patient visits. In this case, the physical exam resulted in the diagnosis of a carotid body tumor (CBT) prior to the patient becoming symptomatic from it. Further work-up was initiated, beginning with a computed tomography-angiogram, which revealed bilateral CBTs. He was referred to the vascular surgery service and underwent surgery to have this mass resected. The right CBT was resected without requiring vascular reconstruction and without postoperative complications. The case depicts the importance of a strong primary care system which becomes even more important in the rural or semi-urban setting and access to a vascular surgeon for appropriate, timely care.

## Introduction

Carotid body tumors (CBTs) develop from neural crest cells like other paragangliomas. They are generally benign, but malignant forms are seen on rare occasions.

The incidence of CBTs is one in 30,000 people and may be familial, sporadic, or hyperplastic. Patients with chronic obstructive pulmonary disorder, those living at high altitudes, and patients with cyanotic heart disease may develop the hyperplastic form [1]. While generally not malignant, CBTs tend to be aggressive locally and involve adjacent nerves. Early resection is recommended to minimize the risk of neural injury, which increases with size [2].

Most tumors present as a neck lump, with or without pressure symptoms like neck pain, dysphagia, dysphonia, cranial nerve palsies, and jaw stiffness [1]. The key is to identify and diagnose these tumors before they are large enough to cause such pressure symptoms to allow for easier resection. In our case, the patient underwent a physical exam at his primary care physician’s office, where appropriate imaging was ordered, a diagnosis was established, and a referral to a vascular surgeon was made.

## Case presentation

We present the case of a 61-year-old male with a past medical history of hypertension. He is a lifetime non-smoker. He presented to his primary care physician for his yearly check-up with no complaints. During the physical exam, the physician noted a 2 x 4 cm pulsatile mass in the right lateral neck at the angle of the mandible. The mass was non-tender, firm, and mobile in the horizontal plane but not in the vertical plane. The patient denied symptoms of dysphagia, neck stiffness, neck pain, odynophagia, dysphonia, or odynophagia. He had no cranial nerve deficits on the exam. 

**Figure 1:**
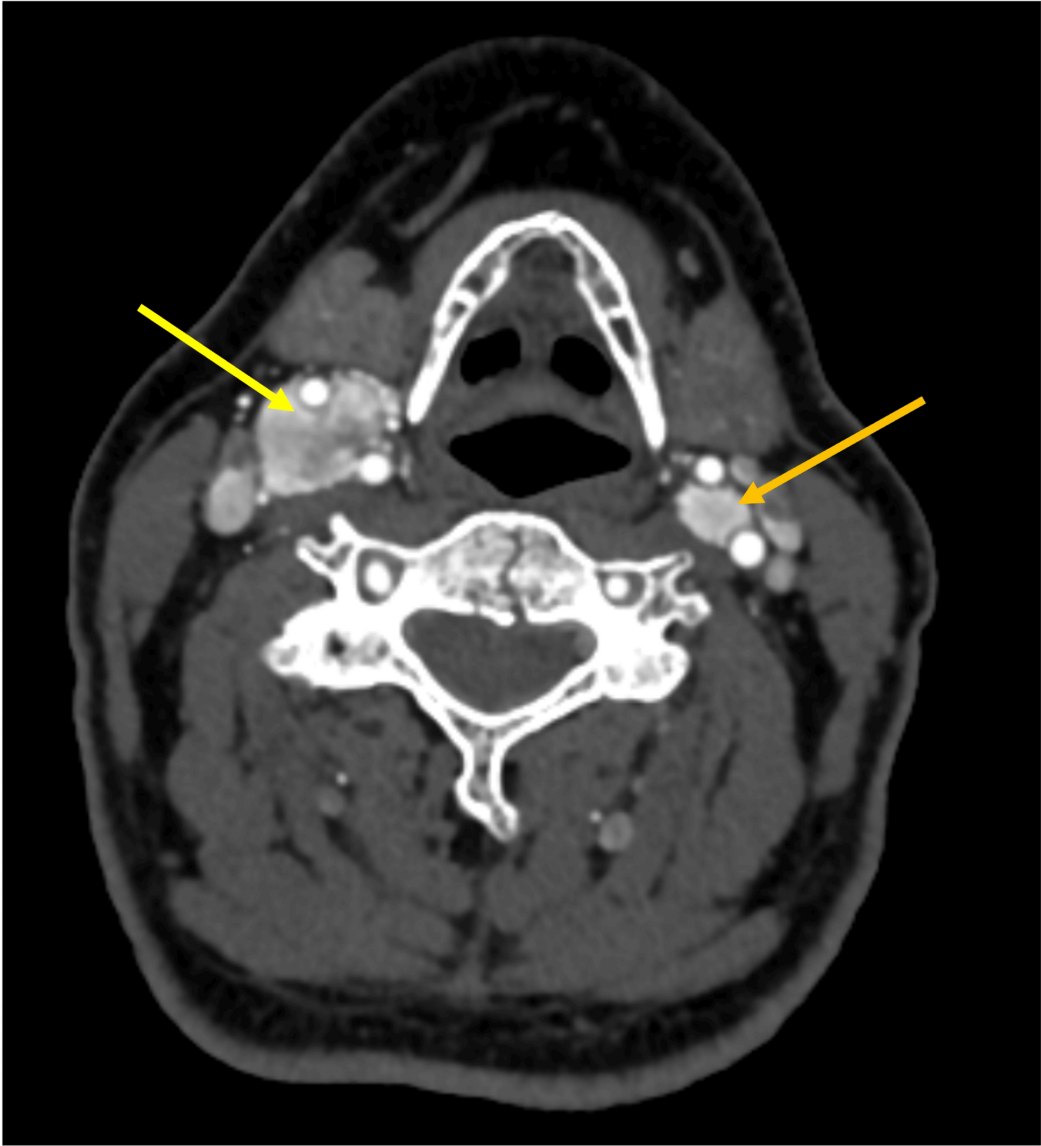
CT angiogram of the neck: Axial section at the level distal to the bifurcation depicting the bilateral carotid body tumors encasing the internal and external carotid arteries on the right (yellow arrow) and abutting the arteries on the left (orange arrow).

**Figure 2:**
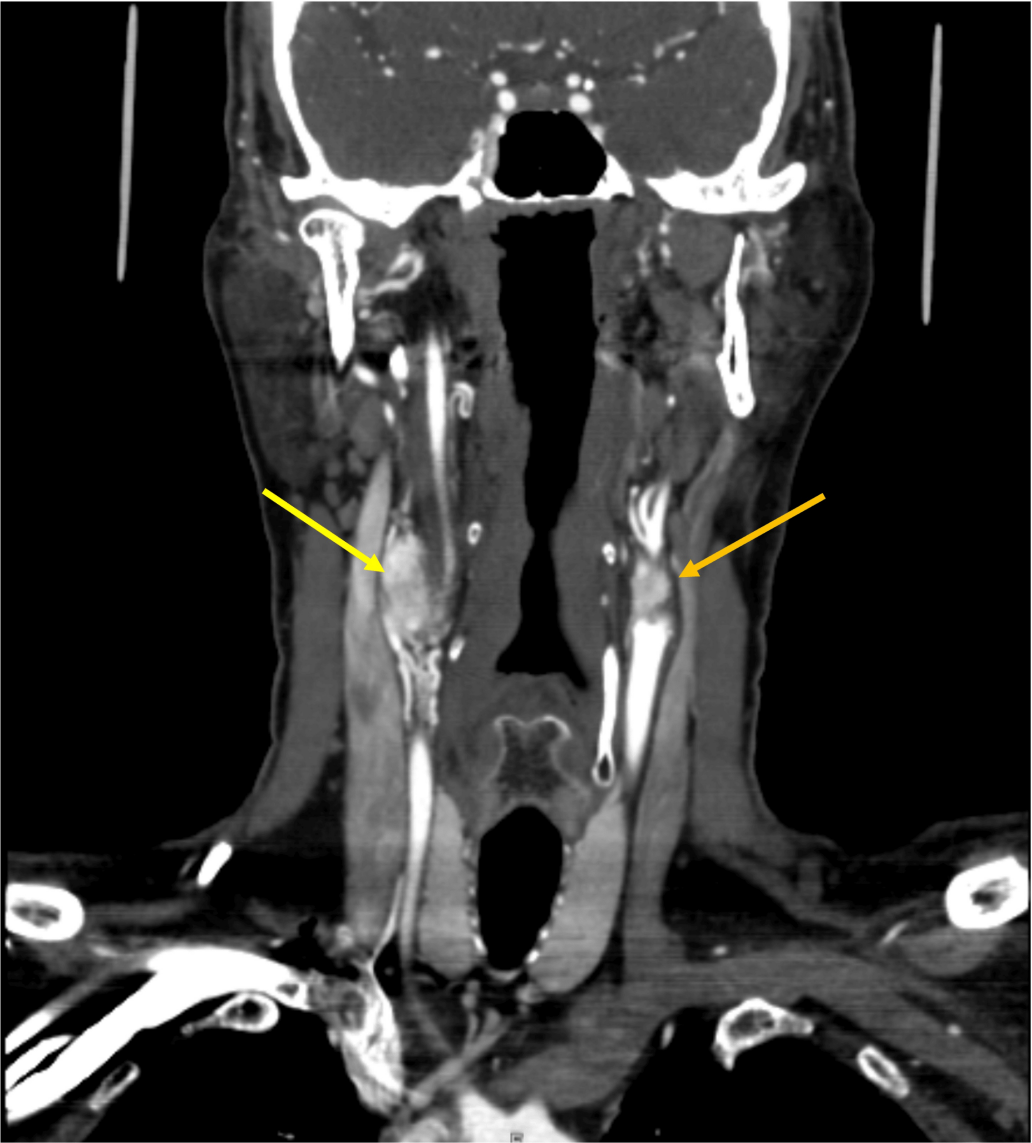
CT angiogram of the neck: Coronal section depicting the bilateral carotid body tumors encasing the internal and external carotid arteries on the right (yellow arrow) and abutting the arteries on the left (orange arrow).

A computed tomography angiogram was ordered, which revealed a 1.8 x 1.8 x 3.4 cm enhancing mass on the right at the carotid bifurcation and a 1.0 x 1.0 x 1.6 cm mass on the left at the carotid bifurcation. On imaging, the tumor on the right appeared to be either a Shamblin group 2 or 3, and the tumor on the left was a Shamblin 2 (Figures 1-2). He was referred to the vascular surgery service and resection was planned, starting with the right tumor first, given its larger size and Shamblin classification.

**Figure 3:**
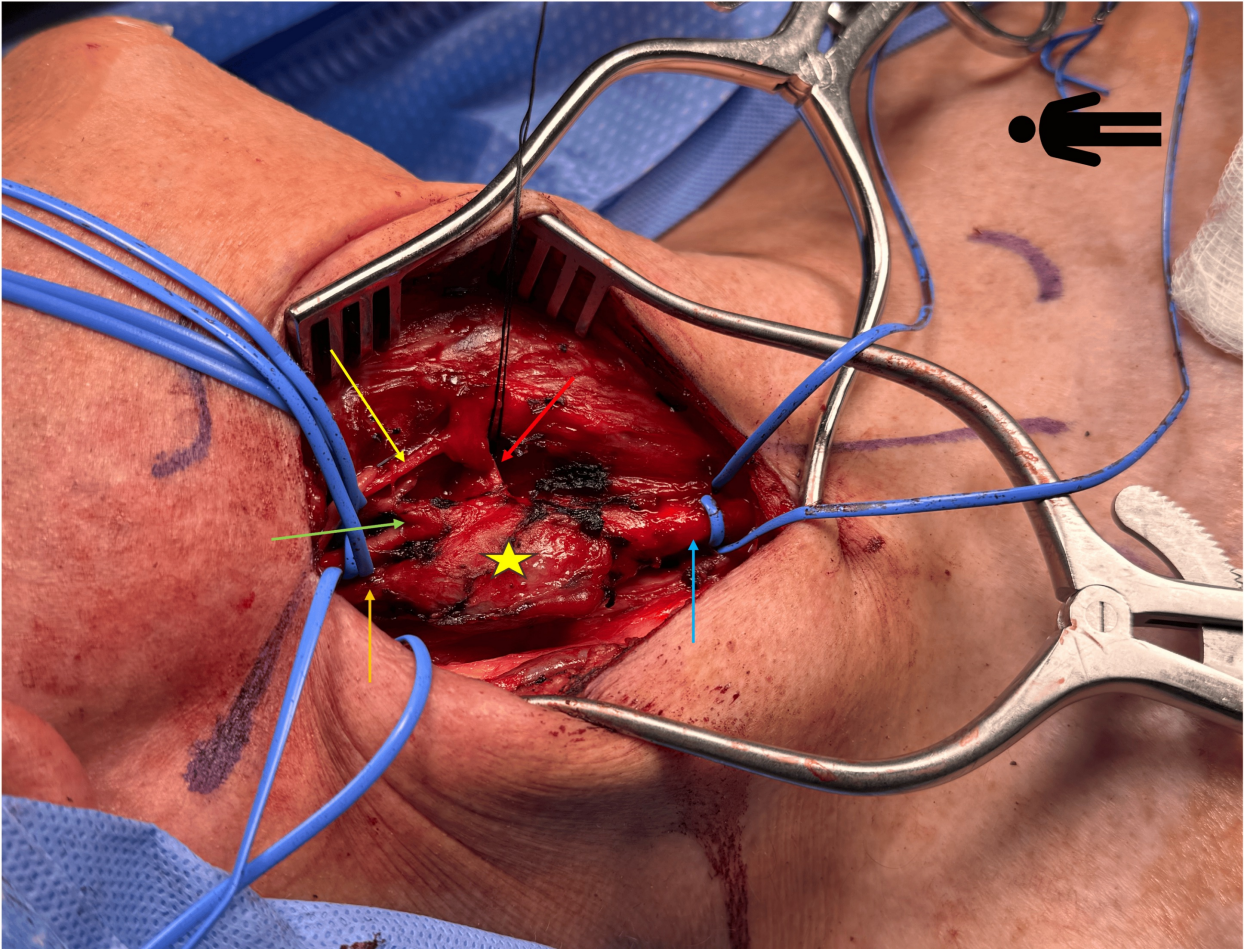
Intraoperative picture with right neck dissection depicting the carotid body tumor (yellow star), the common carotid artery (blue arrow), the internal carotid artery (orange arrow), the external carotid artery (green arrow), and the superior thyroid artery (red arrow).

**Figure 4:**
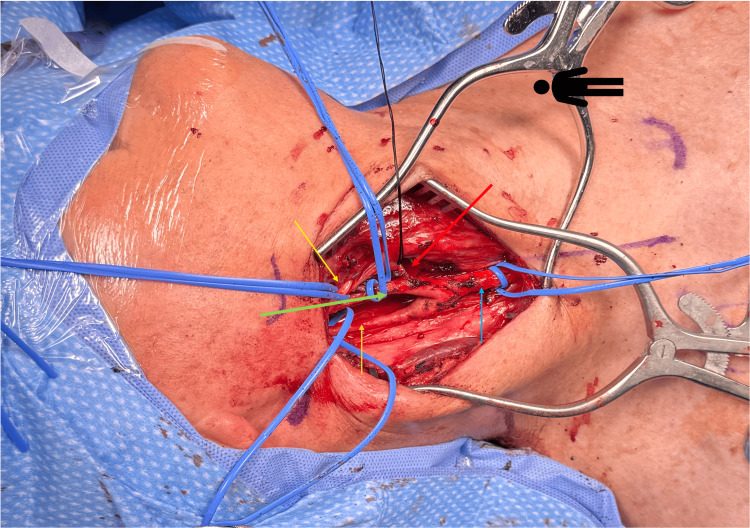
Intraoperative picture with right neck dissection following resection of the carotid body tumor depicting the common carotid artery (blue arrow), the internal carotid artery (orange arrow), the external carotid artery (green arrow), and the superior thyroid artery (red arrow).

**Figure 5:**
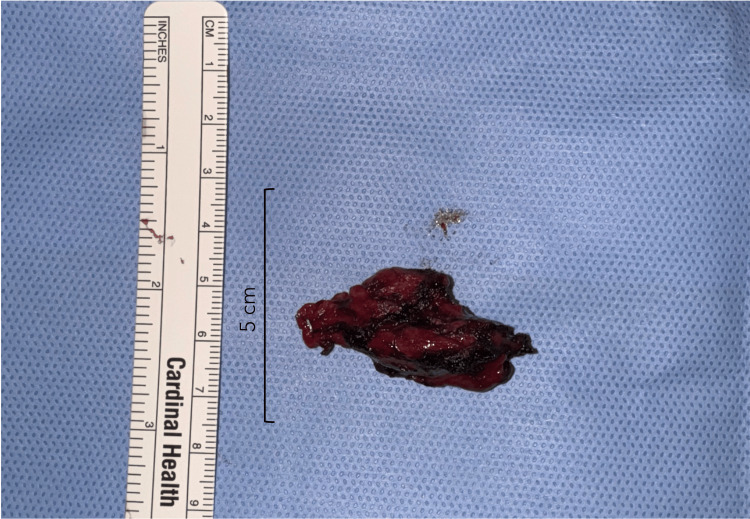
Resected specimen of carotid body tumor, with the scale depicting 5 cm.

Intraoperatively, the patient was placed supine, with the head turned to the left, and a linear incision was made along the anterior border of the sternocleidomastoid under general anesthesia. Dissection was carefully carried down passed the platysma, protecting the internal jugular vein, and ligating and dividing the facial vein. On entering into the carotid sheath, a homogenous hypervascular tumor was evident at the carotid bifurcation, encasing the bifurcation, the internal and external carotid artery (Figure 3). It appeared that the vagus nerve had been drawn into this mass as well. The CBT was classified as a Shamblin group 3 tumor. With delicate dissection, proximal and distal control was obtained at the common carotid, internal carotid, external carotid, and superior thyroid artery. The tumor was then peeled off the arteries and the vagus nerve using sharp dissection and bipolar cautery (Figures 4-5). We ensured that the hypoglossal nerve was identified and protected as well. No clamping of the carotids was required. On emerging from anesthesia, the patient had no focal or cranial nerve deficits. The patient did well postoperatively and was started on a regular diet. He was discharged home on postoperative day one. 

He was seen in the clinic two weeks postoperatively and was found to have no neurological deficits, and the surgical site was found to be healing well with no signs of infection. He is scheduled to undergo resection of the contralateral tumor two months later. Pathology confirmed the tumor to be a paraganglioma. We also recommended his family undergo screening given the bilateral CBTs. 

## Discussion

The first reported excision of a CBT was in 1880 by Riegner [3], where the patient did not survive. Maydl resected a CBT in 1886 [4], with resulting aphasia and hemiplegia in the patient. Albert excised a CBT successfully without ligating the carotid vessels in 1889 [5]. Scudder is credited with the first successful removal of CBT in the United States in 1903 [4]. 

CBTs, while rare, are the most common form of head and neck paragangliomas. The carotid body is a chemoreceptor located at the common carotid artery bifurcation that exerts control on respiration, blood pressure, and heart rate. The glomus cells of the carotid body are derived from neural crest cells, as are other paragangliomas [2,6]. CBTs may be sporadic, familial, or hyperplastic. In the case of familial CBTs or bilateral tumors, screening family members and ongoing surveillance for metachronous tumors is essential [2].

A review of several case series showed the mean age at presentation to be between 45 and 55 years old, with a slight female predilection. Tumors were found to be bilateral in 17% of cases in one study and 36% in another. A mass or lump in the neck was the most common presenting symptom, found in over two-thirds of the patients. These were sometimes associated with pain or pressure symptoms such as dysphagia, dysphonia, odynophagia, dyspnea, or cranial nerve palsies [1,2,7]. Kruger et al. showed that 10% of their patients had vagal nerve palsies, all of which were vagal nerve palsies [2]. On the other hand, Sajid et al. in their study of 91 cases found cranial nerve palsies in 22% of their patients but did not further delineate the nerve involved [1].

Duplex ultrasound and computed tomography (CT) were the most common diagnostic modalities used to evaluate CBTs. Imaging typically depicts a growth at the carotid bifurcation causing splaying of the arteries, often known as the “lyre sign." Additional imaging modalities to further define the hypervascularity and extent are CT angiograms, magnetic resonance imaging, and magnetic resonance angiograms. Conventional angiograms were used in the past but are not used for preoperative planning unless considerations are made for preoperative embolization [1,2]. 

The Shamblin classification is widely used to group CBTs. Group 1 tumors are small and only minimally attached to carotid vessels. These can generally be excised without difficulty. Group 2 tumors are larger and have moderate arterial attachment. They require careful surgical removal. Group 3 tumors are large and tend to incarcerate the bifurcation, common, internal, and external carotid arteries. These are difficult to resect and often require vascular reconstruction [7]. It is essential to realize however, that this classification is difficult to establish solely based on imaging. The extent of vessel adherence can reliably be assessed only intraoperatively. Luna-Ortiz et al. classified 72 patients with CBTs based on the Shamblin classification and compared their surgical time, bleeding, neurological damage, and vascular damage. Based on this, they suggest a modification to the Shamblin classification wherein the size of the tumor (less or greater than 4 cm) and the extent of adherence to the carotid vessels (none, partially, and intimately) played a role in predicting the ease of excision [8].

Several small series have looked at the benefits of preoperative embolization to improve surgical outcomes with regard to blood loss and nerve injury. A large meta-analysis by Abu-Ghanem et al. looked at 15 studies with a total of 470 patients looked to clarify the role of preoperative embolization in patients undergoing CBT resection. The analysis revealed no significant differences in estimated blood loss, operative time, or length of stay. While embolization was associated with a greater risk for cranial nerve injury, possibly secondary to the increased inflammation and edema known to follow embolization, and reduced risks for vascular injury and stroke, these were not statistically significant [9]. This meta-analysis has its limitations given it included studies that were retrospective and small. If embolization is considered, careful evaluation of the individual feeding vessels via angiography is essential. Embolization should be undertaken only if these vessels can be selectively catheterized with proof that there is no reflux of contrast into the internal carotid artery to decrease the risk of stroke [10].

Shamblin et al. described a case series of 90 patients with CBTs requiring surgery. The procedures ranged from resection of the CBT alone, to resection with ligation of the carotid arteries to varying degrees (common, internal, and external carotid arteries). The complications most frequently noted were injury or ligation of the internal jugular vein and injuries to the cranial nerves. The vagus nerve was injured in 15 patients and the hypoglossal nerve injured in 17. These injuries were more frequent because the prevalent thought was that these tumors were malignant. Mortality associated with surgery was 5.7% [7]. Kruger et al. described a craniocaudal approach in their patients to avoid the arterial plexus present at the bifurcation and the ascending pharyngeal branch of the external carotid artery that feeds the CBT. They stressed the importance of the identification and preservation of neural structures. Cranial nerve injuries in this series were lower at 12%. This series had no operative deaths. The malignancy rate was found to be 15% [2]. A third study by Sajid et al. looked at 95 cases from 21 vascular units in the United Kingdom. Cranial nerve deficit on postoperative day 1 was 19%, which reduced to 3% after six months. Surgical mortality was 1% and transient ischemic attack and stroke rate was 1% [1]. 

As experience with carotid surgery has improved, the rates of vascular injury, cranial nerve injury, stroke, and mortality have decreased.

## Conclusions

Our case highlights first and foremost the importance of a thorough physical exam. It is our patient's primary care visit and a physical exam that prompted appropriate testing, which revealed the bilateral CBTs. Despite being a Shamblin group 3 tumor on the right, the patient underwent resection without complication. He will undergo resection of the group 2 tumor on the right subsequently.

The case depicts the importance of a strong primary care system, which becomes all the more important in the rural or semi-urban setting, and access to a vascular surgeon for appropriate, timely care.
